# More about chest physiotherapy and ventilator-associated pneumonia prevention

**DOI:** 10.4103/0972-5229.76090

**Published:** 2010

**Authors:** George Ntoumenopoulos

**Affiliations:** **From:** Guys’ and St Thomas’ NHS Foundation Trust, Westminster Bridge Road, London, UK

Dear Editor,

I read with interest the recent publication by Pattanshetty and Gaude on the role of chest physiotherapy for the prevention of ventilator-associated pneumonia (VAP).[1] This randomized controlled trial is an important addition to the evidence base for non-pharmacological measures to reduce VAP and deserves further comment.

Pattanshetty and Gaude report on the clinical evolution of the clinical pulmonary infection score (CPIS) after intubation and mechanical ventilation, as a surrogate measure of VAP. The authors, however, did not formally report an actual VAP rate (CPIS > 6) for the study. The CPIS has limited sensitivity and specificity and hence may not be the most suitable measure to estimate VAP.[2] Chest physiotherapy for VAP prevention and/or treatment is supported by the evidence that intubation and mechanical ventilation cause airway secretion retention and result in VAP.[3] However, the evidence base for chest physiotherapy for the prevention of VAP is inconsistent.[4] VAP prevention strategies focus on minimizing risk from the aero-digestive tract colonization and oropharyngeal aspiration recommending 45° head-up positioning and chest physiotherapy is not recommended.[5] However, head-down positioning (common component of chest physiotherapy) can facilitate airway secretion clearance and assist to prevent VAP, whereas controversially head-up positioning may impair airway mucus clearance and cause VAP.[6] This supports the role of chest physiotherapy for VAP prevention but requires further clinical confirmation.

The authors should have investigated the impact of other known risk factors associated with VAP such as chronic obstructive pulmonary disease chronic obstructive pulmonary disease (COPD), airway intubation, mechanical ventilation time, intracranial monitoring, airway re-intubation, use of positive end expiratory pressure (PEEP), steroid use, tracheostomy, reduced conscious state and admission acute physiology and chronic health evaluation (APACHE) II score.[7] Considering that there were differences in mortality rates between the two groups,[1] this indicates there may have been group differences (e.g. APACHE II) that have not been accounted for. To improve trial transparency, the authors also should have used the Consort Style of reporting. There are referencing inaccuracies in the body of the text (Jessica *et al*. should be Choi *et al*.). The authors also neglected to report on the microbiological results of the sputum samples, which would have provided a more valid and specific diagnosis of VAP.[1]
